# Systematic Review and Meta-Analysis of Statin Use and Mortality, Intensive Care Unit Admission and Requirement for Mechanical Ventilation in COVID-19 Patients

**DOI:** 10.3390/jcm11185454

**Published:** 2022-09-16

**Authors:** Ut-Sam Lao, Chak-Fun Law, Daniel T. Baptista-Hon, Brian Tomlinson

**Affiliations:** 1 Center for Biomedicine and Innovations, Faculty of Medicine, Macau University Science and Technology, Taipa, Macau SAR 999078, China; 2 Division of Systems Medicine, School of Medicine, University of Dundee, Dundee DD1 4HN, UK

**Keywords:** COVID-19, intensive care unit, mechanical ventilation, meta-analysis, mortality, statin, systematic review

## Abstract

There is mounting evidence that statin use is beneficial for COVID-19 outcomes. We performed a systematic review and meta-analysis to evaluate the association between statin use and mortality, intensive care unit (ICU) admission and mechanical ventilation in COVID-19 patients, on studies which provided covariate adjusted effect estimates, or performed propensity score matching. We searched PubMed, Embase, Web of Science and Scopus for studies and extracted odds or hazard ratios for specified outcome measures. Data synthesis was performed using a random-effects inverse variance method. Risk of bias, heterogeneity and publication bias were analyzed using standard methods. Our results show that statin use was associated with significant reductions in mortality (OR = 0.72, 95% CI: 0.67–0.77; HR = 0.74, 95% CI: 0.69, 0.79), ICU admission (OR = 0.94, 95% CI: 0.89–0.99; HR = 0.76, 95% CI: 0.60–0.96) and mechanical ventilation (OR = 0.84, 95% CI: 0.78–0.92; HR = 0.67, 95% CI: 0.47–0.97). Nevertheless, current retrospective studies are based on the antecedent use of statins prior to infection and/or continued use of statin after hospital admission. The results may not apply to the de novo commencement of statin treatment after developing COVID-19 infection. Prospective studies are lacking and necessary.

## 1. Introduction

Coronavirus infectious disease 2019 (COVID-19) continues to place an incredible burden on morbidity and mortality worldwide. Since the declaration of COVID-19 as a global pandemic, over 560 million confirmed cases and over 6.3 million deaths were reported (data from COVID-19 Data Repository by the Center for Systems Science and Engineering at Johns Hopkins University https://github.com/CSSEGISandData/COVID-19, accessed 25 July 2022) [1]. The development of severe COVID-19 is associated with a number of risk factors, such as older age, male sex and the presence of underlying medical conditions such as cardiovascular diseases [2] and diabetes. Severe COVID-19 cases may involve inflammatory cytokine storm (cytokine release syndrome) resulting in multi-organ failure and mortality [3]. Elevated interleukin-6 (IL-6), a mediator of cytokine release syndrome, has been demonstrated in severe COVID-19 patients [4]. Indeed, diabetic patients have significantly higher serum inflammatory biomarkers including IL-6, C-reactive protein (CRP), D-dimer, serum ferritin and coagulation indices, resulting in a higher chance of an inflammatory storm and eventually worsening the outcome of COVID-19 [5]. Statins are 3-hydroxy-3-methylglutaryl coenzyme A reductase inhibitors used in patients with cardiovascular disease or diabetes to reduce low-density lipoprotein cholesterol. Statins also have other effects, such as antitumor, antioxidative and anti-inflammatory effects [6]. In particular, clinical trials have shown that statins can reduce the levels of CRP [7,8], which may mitigate the severity of the cytokine storm seen in COVID-19 patients.

Since the beginning of the COVID-19 pandemic, a remarkable number of studies, most of them retrospective, have examined the association between statin use and various clinical outcomes in COVID-19. A number of meta-analyses of these retrospective studies have been performed, and the findings are mixed [9,10,11,12,13,14,15,16,17,18,19,20,21,22]. The majority of these focused on mortality as the outcome. Interestingly, meta-analyses that used covariate-adjusted effect estimates found a significant benefit of statins [10,13,14,15,17,21,22,23]. Similarly, one meta-analysis that included propensity score-matched subjects also found a significant benefit of statins on COVID-19 outcomes [22]. This is hardly surprising since the development of severe COVID-19 and mortality are strongly associated with a number of risk factors. Therefore, retrospective studies that report effect estimates of statins that take into account these covariates are less likely to be confounded. Some meta-analyses have evaluated outcomes involving severe COVID-19 (e.g., ICU admission and mechanical ventilation) [12,14,17,19,23,24,25]. The findings are also mixed, since many of these meta-analyses used a mixture of unadjusted and adjusted effect estimates. However, those which focused on studies that adjusted for covariates have found a significant effect of statins on ICU admission or the development of severe COVID-19 [14,17,23]. Indeed, one study specifically evaluated the influence of covariate adjustment and found a significant improvement in the effect estimate of statins on mortality, ICU admission and mechanical ventilation [17].

In light of all the information available, and the availability of additional studies, we performed a systematic review and meta-analysis of the literature to evaluate the association between statin use and COVID-19 mortality, ICU admission and mechanical ventilation. We only analyzed studies reporting covariate-adjusted point estimates, or those which performed propensity score matching on the study population.

## 2. Materials and Methods

This systematic review and meta-analysis were performed and reported according to the Preferred Reporting Items for Systematic Reviews and Meta-Analysis (PRISMA) 2020 guidelines [26].

*Search Strategy*—The information sources used included the PubMed, Embase, Web of Science and Scopus databases. Published articles up to 22 April 2022 were included. The detailed search strategy is outlined in Supplementary Table S1. Reference lists of included articles were also hand searched to identify additional studies. The language of the article screened was limited to English only.

*Eligibility Criteria*—We included retrospective observational studies (e.g., cohort, case-control and case-series studies) in this systematic review. Letters with sufficient detail to evaluate quality and bias were included. The language of the included articles was restricted to English. Case reports, reviews, conference abstracts and editorials were excluded. 

The study population must include patients with confirmed COVID-19, with or without statin use. Studies with no clearly defined control group (i.e., COVID-19 patients not on statins) were excluded. Studies were included irrespective of statin type and dosage. We did not limit our inclusion of studies on the basis of the length of study period. Studies with any of the following outcomes were included: mortality, ICU admission and requirement of assisted ventilation during hospitalization. Studies of statins administered specifically for the treatment of COVID-19 were excluded.

Articles identified from the electronic search were accumulated in EndNote 20 (Clarivate, Philadelphia, PA, USA). Any duplicated studies were removed. Two review authors (L.C.F. and L.U.S.) screened the titles and abstracts to identify eligible studies independently. Full-text screen was then performed and reasons for study exclusion were recorded. Any disagreement on study eligibility was resolved by two other review authors (D.T.B-H. and B.T.). For multiple studies published by the same group of authors, we included only the latest study with the largest number of participants. All authors agreed on the studies included in the analysis.

*Data extraction*—The data from all included studies were extracted by two review authors (L.C.F. and L.U.S.) independently using a modified data extraction form from the Cochrane Foundation [27]. We extracted the following information from each article: name of first author, publication year, study country, sample size, population, propensity score matching, age range, sex, comorbidities, before or after hospital admission use of statins, statin type and dosage, mortality, intensive care admission and assisted ventilation. The unadjusted and adjusted effect estimates were also extracted where possible. Disagreements on data extractions were resolved by two other review authors (D.T.B-H. and B.T.). 

*Quality Assessment*—All cohort studies were evaluated using the Newcastle-Ottawa scale (NOS) independently by two review authors (L.C.F. and L.U.S.). Each study was evaluated on a scale of 0–9, where articles with a high risk of bias scored 0–4, moderate risk of bias scored 5–7 and low risk of bias scored 8–9. 

*Statistical Analysis*—Meta-analyses were performed using Review Manager 5.4 (RevMan version 5.4) [28] on extracted data using a random-effects model with an inverse-variance method. Odds ratios (ORs) for mortality, ICU admission and mechanical ventilation from propensity score-matched studies were pooled with studies with adjusted odds ratios (aORs) with their 95% confidence intervals. Similar pooling was performed with hazard ratios (HRs). Unadjusted data without propensity score matching were not included in the meta-analysis. Heterogeneity among studies was evaluated using the Chi-square test, with a threshold set at *p* < 0.10. The extent of heterogeneity was evaluated using the I^2^ statistic, and classified as low (I^2^ < 30%), moderate (I^2^ = 30–60%) and high (I^2^ > 60%). For analyses with more than 10 studies, funnel plots were constructed for the evaluation of publication bias. Asymmetry in funnel plots was analyzed using Egger’s regression. Sensitivity analyses were performed by observing the effect of removing single, or groups of studies on the overall pooled estimate and heterogeneity.

## 3. Results

### 3.1. Literature Search and Study Characteristics

**Figure 1:**
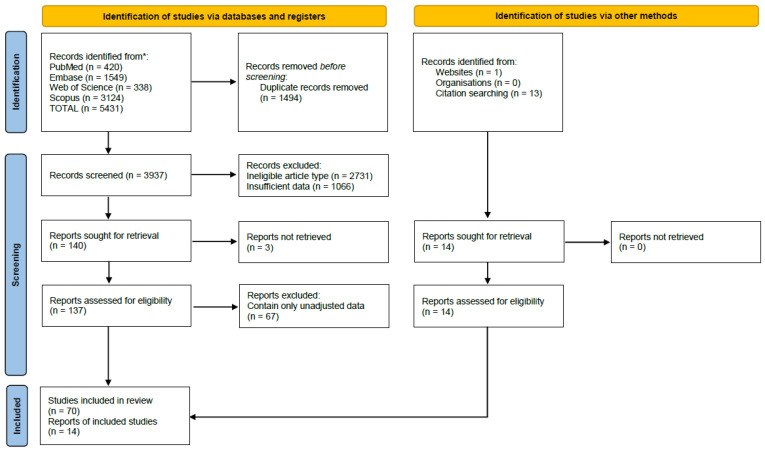
PRISMA flowchart for study selection.

Using the search strategy outlined in the Methods Section and in the Supplemental Information, we identified a total of 5431 articles (PubMed—420, Embase—1549, Web of Science—338 and Scopus—3124; flowchart is shown in Figure 1). Furthermore, 14 additional articles were identified from other journals and hand searching. Following the removal of duplicates, we performed a title and abstract screen on 3937 studies. We applied the eligibility criteria outlined in the Methods Sections and found 2731 studies were not of the correct type, 708 studies did not observe statin use for hospitalized COVID-19 patients, 328 studies did not focus on our interested outcomes, 15 studies contained combined outcome data, 15 studies did not focus on COVID-19 patients and 67 studies contained neither adjusted results nor propensity score matched data. A total of 84 studies with aOR, aHR and/or propensity score-matched results were eligible for data extraction and meta-analysis. A summary of the baseline characteristics of the included studies are shown in Supplementary Table S2.

### 3.2. Effect of Statins on Mortality

**Figure 2:**
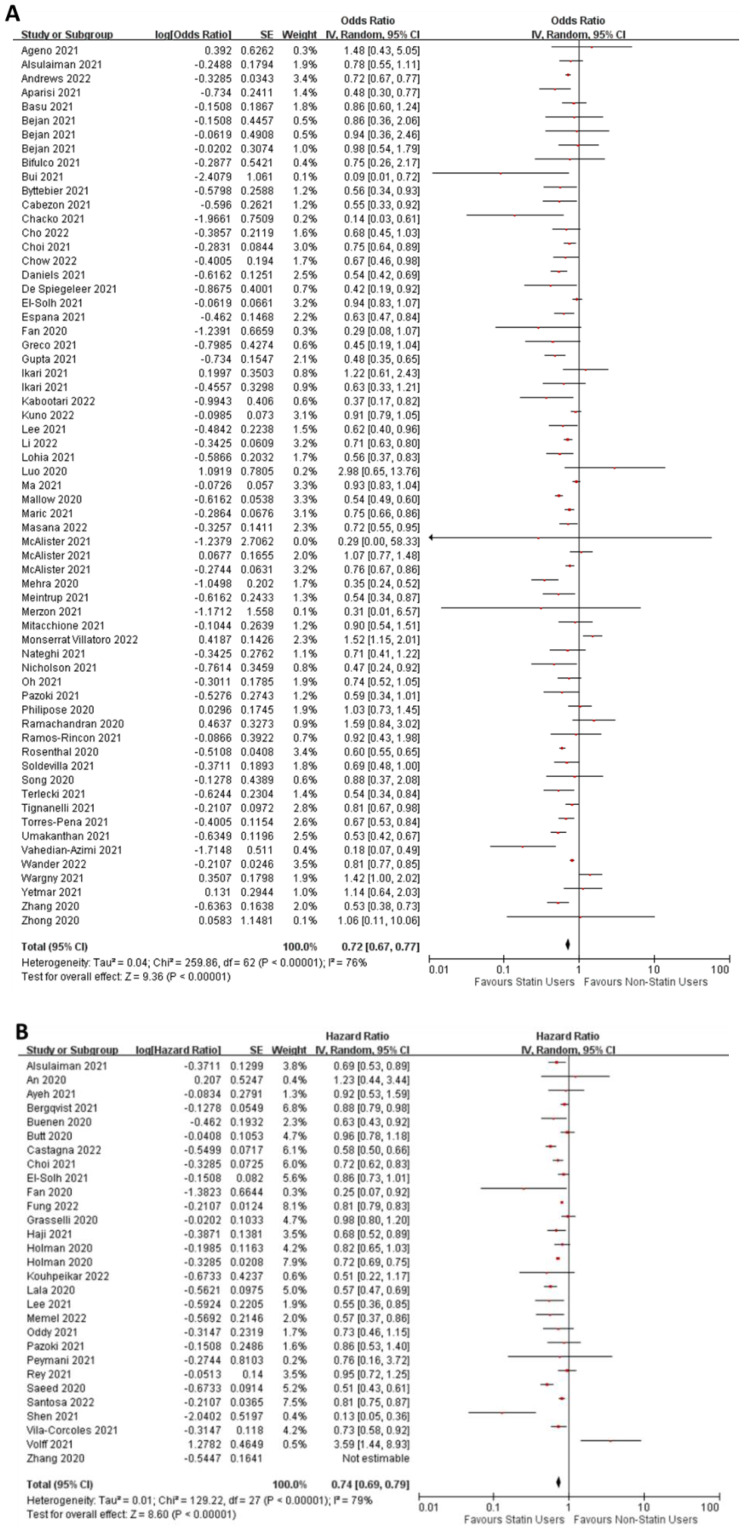
Forest plots of adjusted odds ratio (**A**) and hazard ratio (**B**) for mortality in statin users versus non-users among COVID-19 patients.

*Studies reporting odds ratios*—We included 58 studies with odds ratios corrected by covariate adjustments or propensity score matching between statin users and non-users [25,29,30,31,32,33,34,35,36,37,38,39,40,41,42,43,44,45,46,47,48,49,50,51,52,53,54,55,56,57,58,59,60,61,62,63,64,65,66,67,68,69,70,71,72,73,74,75,76,77,78,79,80,81,82,83,84,85]. Our quality assessment of these studies using the NOS revealed that all studies were of good quality, with a minimum score of 6 (range 6–9; Supplementary Table S3). The pooled estimate of odds ratio was 0.72 (95% CI: 0.67–0.77), and the Z-test revealed a statistically significant pooled odds ratio (Z = 9.36; *p* < 0.00001; Figure 2A). Our meta-analysis therefore showed that the risk of mortality for statin users was lower than non-users. We evaluated the possibility of publication bias in our meta-analysis using funnel plots of the standard error of the logarithmic odds ratio plotted against the odds ratio, followed by an Egger’s regression (Supplementary Figure S1A). We found no significant asymmetry, suggesting there was no publication bias. Heterogeneity existed between the studies (I^2^ = 76%). Visual inspection of individual point estimates and their 95% CI suggest that some studies show statins use increase mortality. We performed a sensitivity analysis to see if individual studies contributed to the overall pooled effect or heterogeneity. We found no effect of sequentially removing individual studies on either the pooled estimate (Supplementary Figure S2), or extent of heterogeneity (data not shown). Our meta-analysis contained odds ratios that are either covariate adjusted or propensity score matched or both. We performed a sensitivity analysis of studies with or without propensity score matching (Supplementary Figure S3). We found no qualitative changes in the pooled estimate or heterogeneity.

*Studies reporting hazard ratios*—We included 28 studies with hazard ratios corrected by covariate adjustments or propensity score matching between statin users and non-users [30,41,45,47,53,70,84,86,87,88,89,90,91,92,93,94,95,96,97,98,99,100,101,102,103,104,105,106]. The NOS scores of these studies were in the good category (Supplementary Table S3). The pooled hazard ratio estimate for mortality was 0.74 (95% CI: 0.69–0.79) and was statistically significant (Z = 8.89; *p* < 0.00001; Figure 2B). Our funnel plot and Egger’s regression analysis found no evidence of publication bias (Supplementary Figure S1B). We also found heterogeneity in this meta-analysis (I^2^ = 79%). Using the same approach as for the mortality OR above, we performed sensitivity analyses of individual studies and found no outliers (Supplementary Figure S4). Sensitivity analyses of studies with or without propensity score matching also revealed no qualitative effect on the pooled estimate or heterogeneity (Supplementary Figure S5). Taken together, our data therefore show that the statin users are less likely to die from COVID-19 but substantial unexplained heterogeneity exists in the data, suggesting that other factors may contribute to determine the effect of statins on COVID-19 patients. The one study showing a significant increase in mortality with statin treatment was in patients already receiving invasive mechanical ventilation [106].

### 3.3. Effect of Statins on ICU Admission

**Figure 3:**
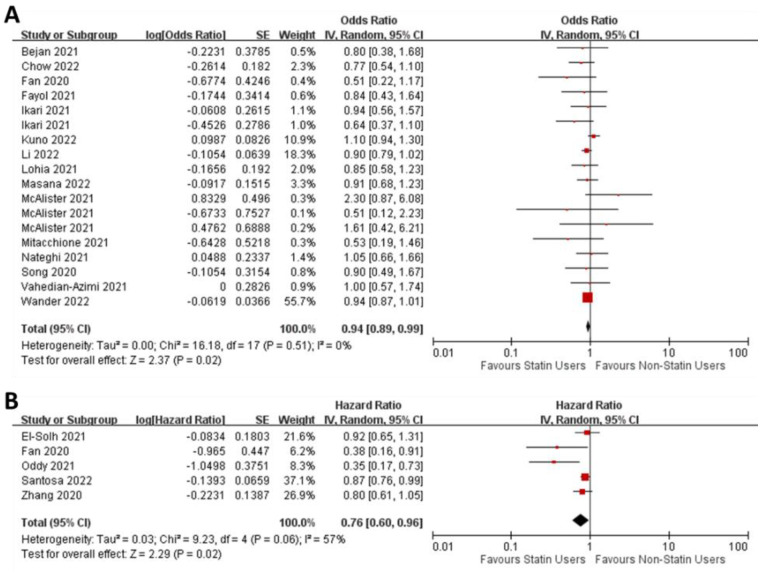
Forest plots of adjusted odds ratio (**A**) and hazard ratio (**B**) for ICU admission in statin users versus non-users among COVID-19 patients.

*Studies reporting odds ratios*—We included 15 studies [25,34,42,47,50,52,54,55,60,61,65,67,76,81,107] with corrected odds ratios for ICU admission by covariate adjustments or propensity score matching between statin users and non-users. NOS risk of bias analysis show that these studies were in the good category (Supplementary Table S3). The pooled estimate of the odds ratio was 0.94 (95% CI: 0.89–0.99; Figure 3A). The Z-test revealed that statin users are significantly less likely to be admitted to the ICU (Z = 2.37; *p* = 0.02). We found no asymmetry in the funnel plots, suggesting no publication bias (Supplementary Figure S6A). We also found little heterogeneity in the meta-analysis (I^2^ = 7%).

*Studies reporting hazard ratios*—We included five covariate-adjusted or propensity score-matched studies reporting hazard ratios for ICU admission between statin users and non-users [45,47,84,99,103]. NOS risk of bias analysis show that these studies were in the good category (Supplementary Table S3). There was a statistically significant benefit of statin use on ICU admission (pooled estimate = 0.76; 95% CI: 0.60–0.96; Z = 2.29; *p* = 0.02). There was no evidence of publication bias (Supplementary Figure S6B). Some heterogeneity exists in the data (I^2^ = 57%), but the χ^2^ test revealed this was not statistically significant (Figure 3B). Our meta-analysis therefore revealed that statin users with COVID-19 were less likely to be admitted to the ICU.

### 3.4. Effect of Statins on Requirement of Mechanical Ventilaion

**Figure 4:**
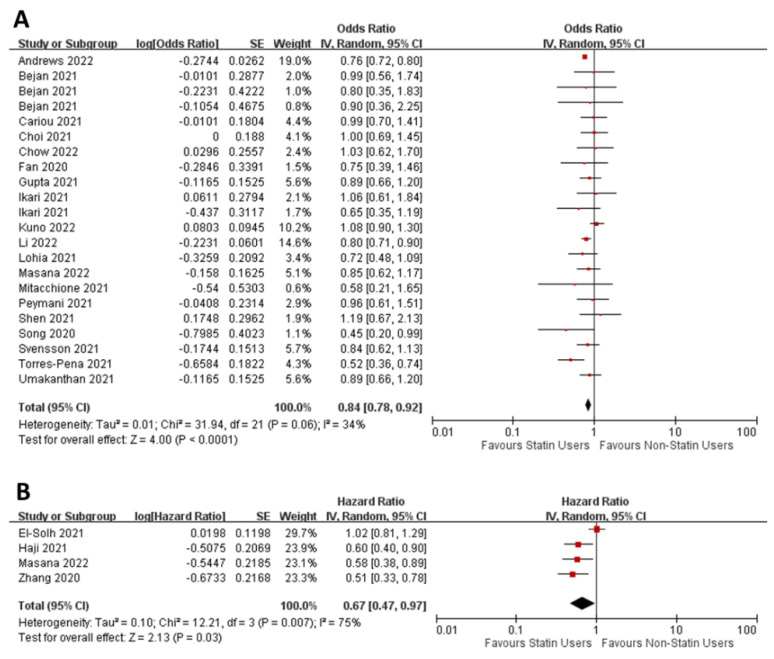
Forest plots of adjusted odds ratio (**A**) and hazard ratio (**B**) for mechanical ventilation in statin users versus non-users among COVID-19 patients.

*Studies reporting odds ratios*—We included 19 studies reporting covariate-adjusted or propensity score-matched odds ratios for requiring mechanical ventilation in statin users versus non-users [31,34,41,42,47,49,50,52,54,55,60,65,76,79,80,100,104,108,109]. NOS risk of bias analysis show that these studies were in the good category (Supplementary Table S3). Our meta-analysis revealed that the use of statins conferred a statistically significant benefit in terms of requiring mechanical ventilation in COVID-19 patients (pooled estimate = 0.84; 95% CI: 0.78–0.92; Z = 4.00; *p* < 0.00001; Figure 4A). There was no evidence of publication bias (Supplementary Figure S7). Some heterogeneity exists in the data (I^2^ = 34%) but is not statistically significant (Figure 4A). 

*Studies reporting hazard ratios*—We included four studies containing covariate-adjusted or propensity score-matched hazard ratio data for mechanical ventilation in statin users and non-users [45,60,84,94]. NOS risk of bias analysis show that these studies were in the good category (Supplementary Table S3). The pooled estimate was 0.67 (95% CI: 0.47–0.97), and there was a statistically significant benefit for statin users (Z = 2.13; *p* = 0.03). The low number of studies precluded the publication bias analyses. Our analysis also revealed heterogeneity in the data (I^2^ = 75%). Visual inspection of the data suggest that one study had a quantitatively different effect to others [45]. We performed a sensitivity analysis by sequentially excluding individual studies from the analysis (Supplementary Figure S8). Our analysis revealed that removing the El-Solh et al. (2021) study from the analysis increased the beneficial effect of statins on mechanical ventilation (0.56; 95% CI: 0.44–0.72) and removed the heterogeneity altogether (I^2^ = 0%). Exclusion of other studies had no effect on heterogeneity. Taken together, our meta-analysis revealed that statin use reduces the requirement of mechanical ventilation in COVID-19 patients.

## 4. Discussion

Our meta-analysis of retrospective observational studies is the largest to date. Our results revealed that statin use was associated with lower mortality, reduced ICU admission and reduced requirement for mechanical ventilation. In terms of mortality, our meta-analysis found an overall 28% and 26% lower mortality in statins users, when pooling adjusted odds ratios and hazard ratios, respectively. For ICU admission, we found that statins users were 6% and 24% less likely to be admitted, when pooling adjusted odds and hazard ratios, respectively. Finally, statin users were 16% and 33% (pooled odds ratio and hazard ratio, respectively) less likely to require mechanical ventilation. We focused our meta-analysis on studies that reported covariate-adjusted point estimates and/or propensity score-matched populations. Nevertheless, we found substantial unexplained heterogeneity in our mortality meta-analysis, which was not surprising given the meta-analysis was based entirely on retrospective studies. This limits the certainty of the results of our mortality meta-analysis. On the other hand, our ICU admission and mechanical ventilation meta-analysis showed little heterogeneity, thus lending further support for the role that statins may play to mitigate severe COVID-19 outcomes.

The beneficial role of statins in the treatment of COVID-19 is supported by preclinical evidence. Statins have been shown to impair the structure, expression and trafficking of CD147 [110,111], which is used by SARS-CoV2 as a co-receptor to infect cells [112]. Statins are also well known to have anti-inflammatory effects, and this has been suggested to contribute to their beneficial effects on cardiovascular outcomes in general [113]. Statins have been shown to regulate the NLRP3 inflammasome [114]. This may be through inhibition of nuclear factor kappa B (NF-kB) and Toll-like receptor 4 [115,116]. This may reduce the excessive cytokine induction mediated by inflammasomes and NF-kB in COVID-19 patients [117].

There have been several meta-analyses evaluating the association between statin use and COVID-19 outcomes. Interestingly, meta-analyses that included both covariate adjusted and unadjusted point estimates in their summary effect analyses have found mixed effects of statins on COVID-19 outcomes [11,12,18,19,24,118]. Those that used covariate adjusted point estimates all reported beneficial effects of statins on COVID-19 outcomes [10,13,14,15,17,21,22,23]. This is not surprising because adverse COVID-19 outcomes have been associated with a wide range of different factors [2]. A recent retrospective study of over 2 million COVID-19 patients in the UK has found factors such as age, male sex, ethnicity, obesity and a number of underlying chronic conditions to be associated with hospital admission and death from COVID-19 [119]. For instance, male patients were approximately 1.5 times more likely to be admitted to hospital and/or die from COVID-19. Obese patients were over twice as likely to be hospitalized. People suffering from hypertension and diabetes were also at significantly increased risk for hospital admission and mortality from COVID-19. Indeed, the use of more than two antidiabetic or antihypertensive drugs was associated with increased mortality from COVID-19 [120]. Therefore, underlying cardiovascular diseases and/or diabetes will have a significant influence on point estimates for mortality and progression to more severe COVID-19 (i.e., ICU admission and requirement for mechanical ventilation). Similarly, mortality and severity point estimates will also be influenced by other confounding factors listed above. We therefore argue that it is crucial to use only multivariable-adjusted point estimates in these meta-analyses. Indeed, the effect of confounding factors are highlighted by the meta-analyses performed by Diaz-Arocutipa et al. (2021) and Scheen (2021) in which the effect of using unadjusted and adjusted point estimates were evaluated. Diaz-Arocutipa et al. (2021) found that the pooled estimate for mortality using unadjusted point estimates was 1.16, while that using adjusted point estimates was 0.67. Importantly, the extent of heterogeneity (assessed by the I^2^ statistic) also reduced from 99% to 79% in the unadjusted and adjusted analyses, respectively. This is also consistent with the findings of Scheen (2021), who directly compared the pooled hazard ratio estimates from univariate (unadjusted) and multivariate (adjusted) analyses and found a statistically significant improvement in the hazard ratio for mortality in the multivariate-adjusted analysis. We also focused our meta-analysis on studies that used propensity score-matched populations. Our sensitivity analysis revealed that the odds ratio or hazard ratio for studies that used propensity score matching was qualitatively and quantitatively similar to those that only reported covariate-adjusted point estimates. Our findings are also consistent with one other systematic review that evaluated propensity score-matched populations [22]. We therefore argue that propensity score matching should always be considered when performing retrospective clinical studies.

Our meta-analysis suffers from a number of limitations. The inclusion of non-randomized retrospective studies will inevitably increase the heterogeneity in our meta-analysis, although such heterogeneity was only observed in the mortality. Our sensitivity analyses did not reveal the causes for this heterogeneity, which limits the certainty in the mortality summary effect estimates. Nevertheless, the heterogeneity for our ICU admission odds and hazard ratio were substantially less (I^2^ = 0% and 57%, respectively), and that for the mechanical ventilation odds ratio was 34%. The substantial heterogeneity seen with the mechanical ventilation hazard ratio was due to one study in particular [45]. This study was based on military veterans and as such was predominantly male (over 90%). Male sex is a known risk factor for the development of severe COVID-19 [2]. It was also not established whether the participants in the statin group in that study were actually taking statin at the time of developing infection with COVID-19. A similar problem exists with many of the studies reporting antecedent statin use. For the unexplained heterogeneity in our mortality meta-analysis, we could speculate on a number of factors. Such heterogeneity could come from the type and dosage of statins used by patients, which may not be clearly stated in some studies. The timing of statin administration may also be a factor. Prior meta-analyses have shown that patients already on statins prior to hospitalization with COVID-19 showed no benefit in terms of mortality, when compared to those where statins were only initiated after hospitalization [9,16]. Many studies we included do not make this distinction, and it can be reasonably assumed that pre-hospitalization statin use would have continued following hospital admission. Furthermore, the duration of statin administration and adherence to this prescription may also show great variation. This is consistent with the high level of heterogeneity in the studies or subgroups that isolated the patients who have been taking statins before hospitalization [9,16]. We therefore believe that studies describing the use of statins after hospitalization provide a more reliable indication that the patient had taken the drug. Another source of variation comes from studies that have evaluated statins as part of a multi-treatment modality for COVID-19, or studies that focused on a particular subgroup of patients (e.g., diabetics, hypertensives or elderly). Finally, heterogeneity will also exist in the criteria for covariate adjustments, propensity score matching and the follow up time for mortality data.

Despite these limitations, our findings are in broad agreement with similar meta-analyses performed, and therefore, the conclusions that can be drawn from our studies are strong and generalizable. Furthermore, the pooled estimates for odds ratios and hazard ratios of our specified outcomes were qualitatively and quantitatively in agreement with each other. Therefore, our findings and those of other well conducted meta-analyses provide strong evidence for the appropriate use of statins to reduce the risk of cardiovascular disease in people who may be at risk of developing COVID-19. Indeed, the use of statins has been advocated for the treatment of Middle East Respiratory Syndrome (MERS) [117,121]. We believe that this is a strong basis on which to conduct well-designed prospective clinical trials to evaluate the de novo use of statins to treat COVID-19. In this study, we excluded retrospective studies on the use of statins specifically for the treatment of COVID-19 because we argue that this can only be appropriately tested using prospective clinical trials. A number of clinical trials are ongoing, and two of these trials have reported their findings. The INSPIRATION-S study is a randomized clinical trial that directly compared the effect of atorvastatin with placebo on mortality and adverse cardiovascular events in 605 COVID-19 patients [122]. The study found no significant differences in the composite outcomes (incidence of mortality and cardiovascular events). However, the authors did note that the incidence of the primary outcome was lower than expected, and therefore, more subtle effects of statins may not be detectable. Another randomized clinical trial compared the effects of emtricitabine, tenofovir, colchicine and rosuvastatin on 28-day mortality in 994 COVID-19 patients [123]. The study reports that while emtricitabine with tenofovir or colchicine with rosuvastatin did not improve mortality, the combination of the four drugs significantly improved 28-day mortality (HR = 0.53, 95% CI: 0.29–0.96), and reduced the requirement for mechanical ventilation (risk difference = 0.08; 95% CI: 0.11–0.04). However, we note that patient recruitment period for both of these trials overlapped with the roll-out of the different COVID-19 vaccines, which had substantial efficacy to prevent severe COVID-19 and death. Neither of these studies took vaccination status into account during randomization and this may have affected the results. Four ongoing randomized clinical trials will seek to directly evaluate the effects of statin use in COVID-19 patients (NCT02735707, NCT04380402, NCT04952350 and NCT04900155). Others are looking at a combination of drugs, including statins (NCT04472611, NCT04813471, NCT04631536, NCT04466241 and NCT04348695). Interestingly, there are two ongoing randomized clinical trials evaluating the effect of statins on post-COVID-19 syndrome (i.e., “long COVID”, NCT04904536 and NCT04801940). We anticipate the results of these ongoing trials with much enthusiasm, and we believe further meta-analysis of the effect of statins on COVID-19 outcomes should focus on randomized clinical trials.

## 5. Conclusions

The results of our meta-analysis have shown that the use of statins is associated with significantly lower risks of mortality, ICU admission and mechanical ventilation in COVID-19 patients. These studies were based on the antecedent use of statins prior to infection or hospital admission and/or the continued use of statin after hospital admission rather than commencement of statin treatment after developing COVID-19 infection. The use of statin treatment to reduce cardiovascular risk in appropriate patients should be encouraged during the COVID-19 pandemic. Whether it may be beneficial to start statin treatment after developing the infection may be difficult to prove with the ongoing trials because of the rapid changes in the use of vaccinations and effective antiviral drugs and the changes in the SARS-CoV-2 variants.

## Supplementary Materials

The following are available online at https://www.mdpi.com/article/10.3390/jcm11185454/s1, Figure S1: Funnel plot and asymmetry analysis for mortality odds and hazard ratio, Figure S2: Sensitivity analysis of individual study effect on mortality odds ratio, Figure S3: Sensitivity analysis of studies with or without propensity score matching effect on mortality odds ratio, Figure S4: Sensitivity analysis of individual study effect on mortality hazard ratio, Figure S5: Sensitivity analysis of studies with or without propensity score matching effect on mortality hazard ratio, Figure S6: Funnel plot and asymmetry analysis for ICU admission odds and hazard ratio, Figure S7: Funnel plot and asymmetry analysis for mechanical ventilation odds ratio, Figure S8: Sensitivity analysis of individual study effect on mechanical ventilation hazard ratio, Table S1: Literature search strategy, Table S2: Summary of included studies, Table S3: Newcastle-Ottawa Scale assessment of study quality.
