# Supplementary material for: Systematic Review and Meta-Analysis of Statin Use and Mortality, Intensive Care Unit Admission and Requirement for Mechanical Ventilation in COVID-19 Patients
Source: J Clin Med. 2022 Sep 16;11(18):5454. doi: 10.3390/jcm11185454 (PMC9501062; doi:10.3390/jcm11185454)
Supplement: Supplementary file 1 [file jcm-11-05454-s001.zip › jcm-1876015-supplementary.pdf]

Systematic review and meta-analysis of statin use and COVID-19 severity outcomes

Supplementary Information

Supplementary Figure S1 – Funnel plot and asymmetry analysis for mortality odds and hazard ratio

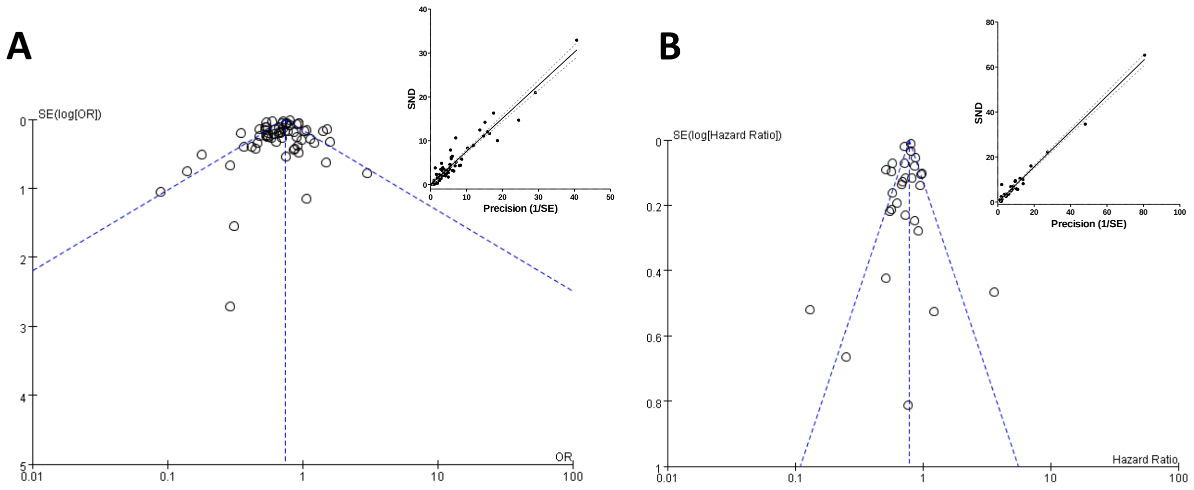

Funnel plots of the standard error of the logarithm of the mortality odds (A) or hazard ratio (B). Vertical dotted lines indicate the overall effect and the slanted dotted lines indicate the 95% confidence interval. Inset shows the Egger's regression of the standard normalised difference (SND) against the precision (1/SE). The solid lines are the non-weighted linear regression and the dotted lines indicate the 90% confidence interval of the linear regression. Our analysis reveal no significant asymmetry in the funnel plots (P = 0.87 for OR and P = 0.73 for HR).

Supplementary Figure S2 – Sensitivity analysis of individual study effect on mortality odds ratio

Study removed:

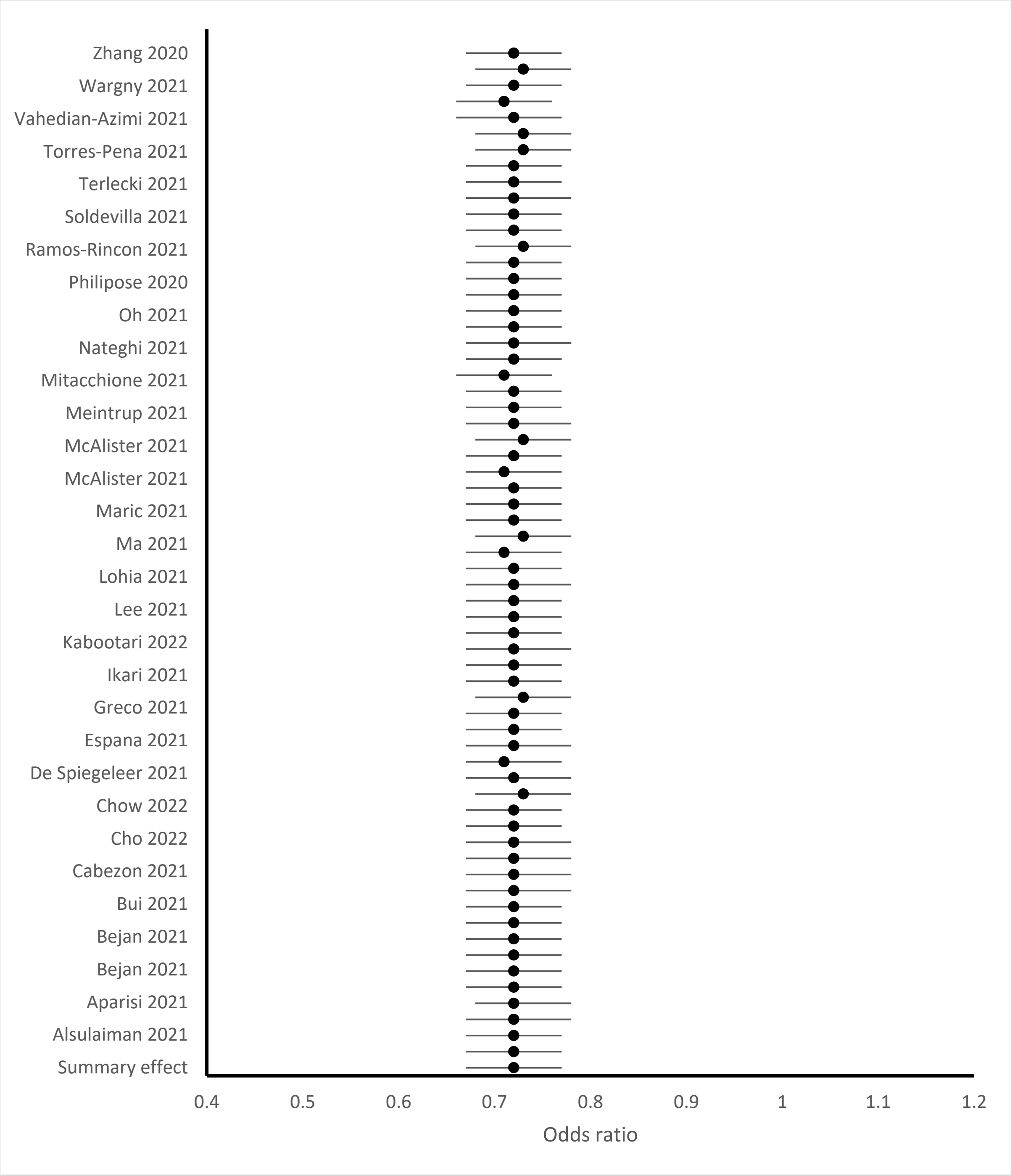

Sensitivity analysis of the data by observing the effect on the overall summary effect (shown at the bottom of the graph) through removing sequential studies. Our analysis reveal that there is no single study the removal of which affects the overall summary effect.

Supplementary Figure S3 – Sensitivity analysis of studies with or without propensity score matching effect on mortality odds ratio

Study or Subgroup	log[Odds Ratio]	SE	Weight	Odds Ratio IV, Random, 95% CI
Ageno 2021	0.392	0.6262	0.5%	1.48 [0.43, 5.05]
Andrews 2022	-0.3285	0.0343	5.8%	0.72 [0.67, 0.77]
Aparisi 2021	-0.734	0.2411	2.4%	0.48 [0.30, 0.77]
Basu 2021	-0.1508	0.1867	3.1%	0.86 [0.60, 1.24]
Bifulco 2021	-0.2877	0.5421	0.7%	0.75 [0.26, 2.17]
Byttebier 2021	-0.5798	0.2588	2.2%	0.56 [0.34, 0.93]
Cabezon 2021	-0.596	0.2621	2.1%	0.55 [0.33, 0.92]
Chacko 2021	-1.9661	0.7509	0.4%	0.14 [0.03, 0.61]
Cho 2022	-0.3857	0.2119	2.7%	0.68 [0.45, 1.03]
Daniels 2021	-0.6162	0.1251	4.2%	0.54 [0.42, 0.69]
De Spiegeleer 2021	-0.8675	0.4001	1.1%	0.42 [0.19, 0.92]
El-Solh 2021	-0.0619	0.0661	5.4%	0.94 [0.83, 1.07]
Espana 2021	-0.462	0.1468	3.8%	0.63 [0.47, 0.84]
Greco 2021	-0.7985	0.4274	1.0%	0.45 [0.19, 1.04]
Ikari 2021	0.1997	0.3503	1.4%	1.22 [0.61, 2.43]
Ikari 2021	-0.4557	0.3298	1.5%	0.63 [0.33, 1.21]
Kabootari 2022	-0.9943	0.406	1.1%	0.37 [0.17, 0.82]
Luo 2020	1.0919	0.7805	0.3%	2.98 [0.65, 13.76]
Mallow 2020	-0.6162	0.0538	5.6%	0.54 [0.49, 0.60]
McAlister 2021	-1.2379	2.7062	0.0%	0.29 [0.00, 58.33]
McAlister 2021	0.0677	0.1655	3.5%	1.07 [0.77, 1.48]
McAlister 2021	-0.2744	0.0631	5.4%	0.76 [0.67, 0.86]
Mehra 2020	-1.0498	0.202	2.9%	0.35 [0.24, 0.52]
Meintrup 2021	-0.6162	0.2433	2.3%	0.54 [0.34, 0.87]
Merzon 2021	-1.1712	1.558	0.1%	0.31 [0.01, 6.57]
Nateghi 2021	-0.3425	0.2762	2.0%	0.71 [0.41, 1.22]
Nicholson 2021	-0.7614	0.3459	1.4%	0.47 [0.24, 0.92]
Oh 2021	-0.3011	0.1785	3.2%	0.74 [0.52, 1.05]
Pazoki 2021	-0.5276	0.2743	2.0%	0.59 [0.34, 1.01]
Philipose 2020	0.0296	0.1745	3.3%	1.03 [0.73, 1.45]
Ramachandran 2020	0.4637	0.3273	1.6%	1.59 [0.84, 3.02]
Ramos-Rincon 2021	-0.0866	0.3922	1.2%	0.92 [0.43, 1.98]
Rosenthal 2020	-0.5108	0.0408	5.7%	0.60 [0.55, 0.65]
Song 2020	-0.1278	0.4389	1.0%	0.88 [0.37, 2.08]
Terlecki 2021	-0.6244	0.2304	2.5%	0.54 [0.34, 0.84]
Tignanelli 2021	-0.2107	0.0972	4.8%	0.81 [0.67, 0.98]
Vahedian-Azimi 2021	-1.7148	0.511	0.8%	0.18 [0.07, 0.49]
Wander 2022	-0.2107	0.0246	5.9%	0.81 [0.77, 0.85]
Wargny 2021	0.3507	0.1798	3.2%	1.42 [1.00, 2.02]
Yetmar 2021	0.131	0.2944	1.8%	1.14 [0.64, 2.03]
Zhong 2020	0.0583	1.1481	0.2%	1.06 [0.11, 10.06]
**Total (95% CI)**			**100.0%**	**0.70 [0.64, 0.77]**

Heterogeneity: $Tau^2 = 0.04$; $Chi^2 = 177.62$, df = 40 ($P < 0.00001$); $I^2 = 77\%$
Test for overall effect: Z = 7.40 ($P < 0.00001$)

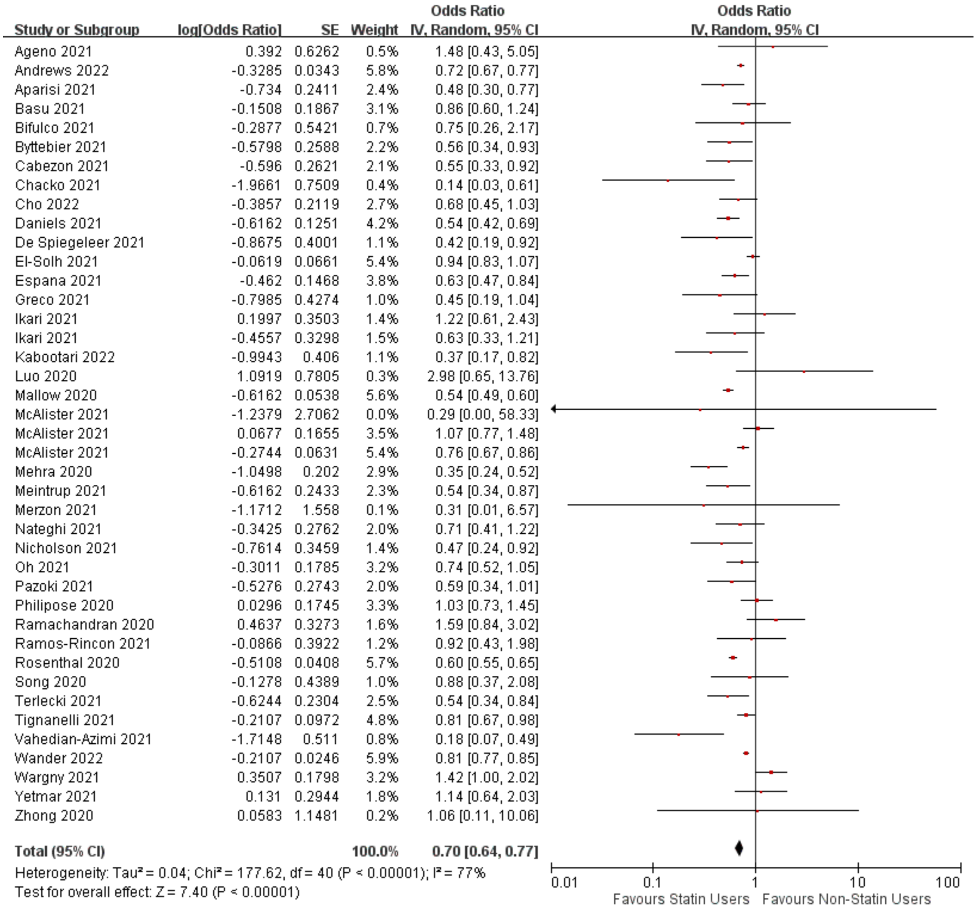

Study or Subgroup	log[Odds Ratio]	SE	Weight	Odds Ratio IV, Random, 95% CI
Alsulaiman 2021	-0.2488	0.1794	4.6%	0.78 [0.55, 1.11]
Bejan 2021	-0.1508	0.4457	1.4%	0.86 [0.36, 2.06]
Bejan 2021	-0.0619	0.4908	1.2%	0.94 [0.36, 2.46]
Bejan 2021	-0.0202	0.3074	2.5%	0.98 [0.54, 1.79]
Bui 2021	-2.4079	1.061	0.3%	0.09 [0.01, 0.72]
Choi 2021	-0.2831	0.0844	7.0%	0.75 [0.64, 0.89]
Chow 2022	-0.4005	0.194	4.2%	0.67 [0.46, 0.98]
Fan 2020	-1.2391	0.6659	0.7%	0.29 [0.08, 1.07]
Gupta 2021	-0.734	0.1547	5.2%	0.48 [0.35, 0.65]
Kuno 2022	-0.0985	0.073	7.3%	0.91 [0.79, 1.05]
Lee 2021	-0.4842	0.2238	3.7%	0.62 [0.40, 0.96]
Li 2022	-0.3425	0.0609	7.5%	0.71 [0.63, 0.80]
Lohia 2021	-0.5866	0.2032	4.1%	0.56 [0.37, 0.83]
Ma 2021	-0.0726	0.057	7.6%	0.93 [0.83, 1.04]
Maric 2021	-0.2864	0.0676	7.4%	0.75 [0.66, 0.86]
Masana 2022	-0.3257	0.1411	5.5%	0.72 [0.55, 0.95]
Mitacchione 2021	-0.1044	0.2639	3.0%	0.90 [0.54, 1.51]
Monserrat Villatoro 2022	0.4187	0.1426	5.5%	1.52 [1.15, 2.01]
Soldevilla 2021	-0.3711	0.1893	4.3%	0.69 [0.48, 1.00]
Torres-Pena 2021	-0.4005	0.1154	6.2%	0.67 [0.53, 0.84]
Umakanthan 2021	-0.6349	0.1196	6.1%	0.53 [0.42, 0.67]
Zhang 2020	-0.6363	0.1638	4.9%	0.53 [0.38, 0.73]
**Total (95% CI)**			**100.0%**	**0.73 [0.65, 0.82]**

Heterogeneity: $Tau^2 = 0.04$; $Chi^2 = 78.51$, df = 21 ($P < 0.00001$); $I^2 = 73\%$
Test for overall effect: Z = 5.48 ($P < 0.00001$)

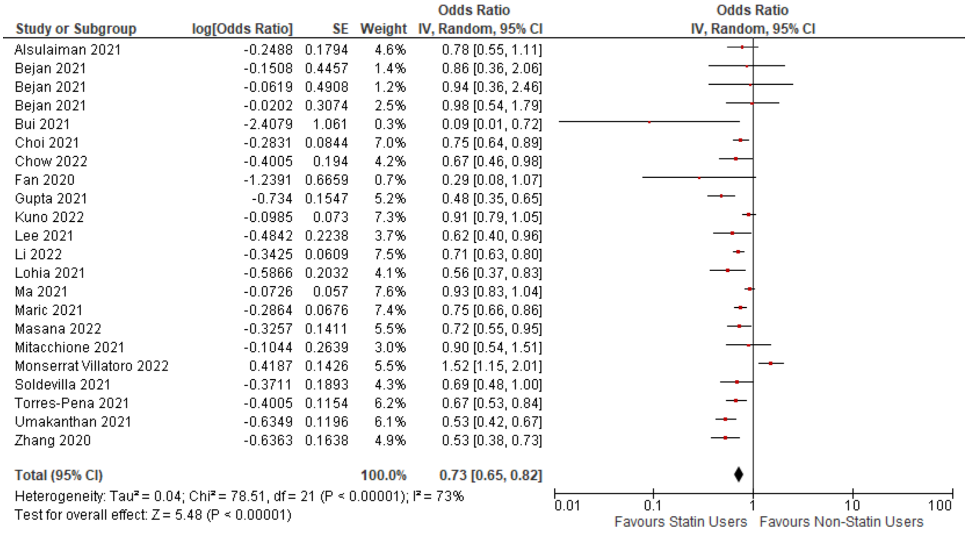

Sensitivity analysis of the mortality odds ratio summary effect by performing separate analysis of studies without (top) or with (bottom) propensity score matching. Our analysis reveal that studies with or without propensity score matching had qualitatively and quantitatively the same effect on the overall estimate. The extent of heterogeneity is also similar in the two groups of studies.

Supplementary Figure S4 – Sensitivity analysis of individual study effect on mortality hazard ratio

Study removed:

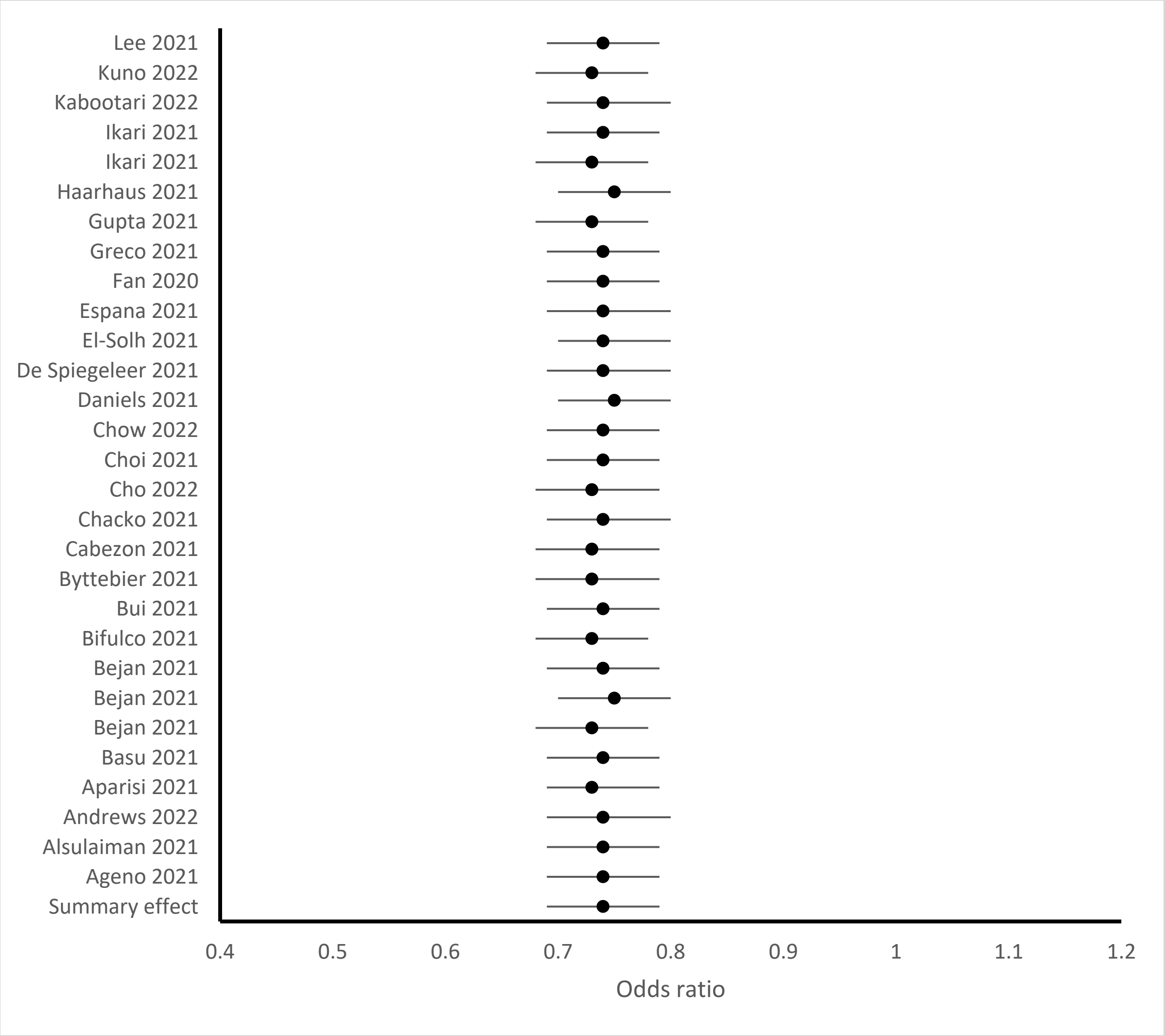

Sensitivity analysis of the data by observing the effect on the overall summary effect (shown at the bottom of the graph) through removing sequential studies. Our analysis reveal that there is no single study the removal of which affects the overall summary effect.

Supplementary Figure S5 – Sensitivity analysis of studies with or without propensity score matching effect on mortality hazard ratio

Study or Subgroup	log[Hazard Ratio]	SE	Weight	Hazard Ratio IV, Random, 95% CI
An 2020	0.207	0.5247	0.8%	1.23 [0.44, 3.44]
Ayeh 2021	-0.0834	0.2791	2.3%	0.92 [0.53, 1.59]
Bergqvist 2021	-0.1278	0.0549	8.5%	0.88 [0.79, 0.98]
Buenen 2020	-0.462	0.1932	3.8%	0.63 [0.43, 0.92]
Butt 2020	-0.0408	0.1053	6.6%	0.96 [0.78, 1.18]
Castagna 2022	-0.5499	0.0717	7.9%	0.58 [0.50, 0.66]
Grasselli 2020	-0.0202	0.1033	6.7%	0.98 [0.80, 1.20]
Haji 2021	-0.3871	0.1381	5.4%	0.68 [0.52, 0.89]
Holman 2020	-0.1985	0.1163	6.1%	0.82 [0.65, 1.03]
Holman 2020	-0.3285	0.0208	9.5%	0.72 [0.69, 0.75]
Kouhpeikar 2022	-0.6733	0.4237	1.1%	0.51 [0.22, 1.17]
Lala 2020	-0.5621	0.0975	6.9%	0.57 [0.47, 0.69]
Memel 2022	-0.5692	0.2146	3.3%	0.57 [0.37, 0.86]
Pazoki 2021	-0.1508	0.2486	2.7%	0.86 [0.53, 1.40]
Rey 2021	-0.0513	0.14	5.3%	0.95 [0.72, 1.25]
Saeed 2020	-0.6733	0.0914	7.1%	0.51 [0.43, 0.61]
Santosa 2022	-0.2107	0.0365	9.1%	0.81 [0.75, 0.87]
Vila-Corcoles 2021	-0.3147	0.118	6.1%	0.73 [0.58, 0.92]
Volff 2021	1.2782	0.4649	1.0%	3.59 [1.44, 8.93]
**Total (95% CI)**			**100.0%**	**0.75 [0.68, 0.83]**

Heterogeneity: Tau² = 0.02; Chi² = 87.95, df = 18 (P < 0.00001); I² = 80%
Test for overall effect: Z = 5.97 (P < 0.00001)

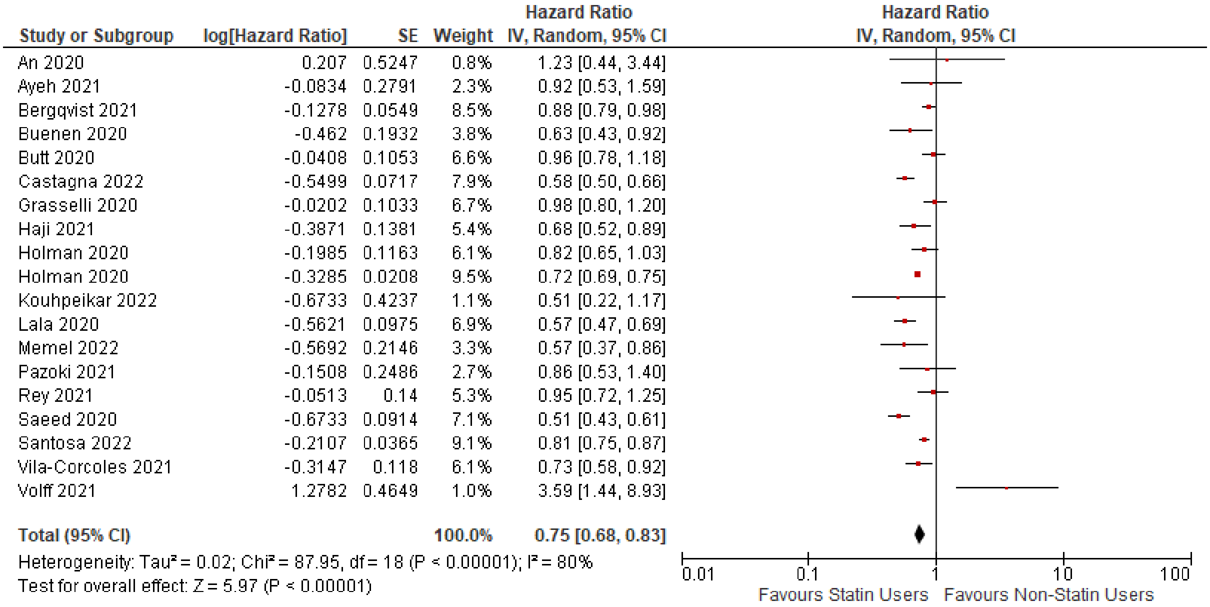

Study or Subgroup	log[Hazard Ratio]	SE	Weight	Hazard Ratio IV, Random, 95% CI
Alsulaiman 2021	-0.3711	0.1299	12.9%	0.69 [0.53, 0.89]
Choi 2021	-0.3285	0.0725	18.9%	0.72 [0.62, 0.83]
El-Solh 2021	-0.1508	0.082	17.8%	0.86 [0.73, 1.01]
Fan 2020	-1.3823	0.6644	1.0%	0.25 [0.07, 0.92]
Fung 2022	-0.2107	0.0124	23.7%	0.81 [0.79, 0.83]
Lee 2021	-0.5924	0.2205	6.9%	0.55 [0.36, 0.85]
Oddy 2021	-0.3147	0.2319	6.4%	0.73 [0.46, 1.15]
Peymani 2021	-0.2744	0.8103	0.7%	0.76 [0.16, 3.72]
Shen 2021	-2.0402	0.5197	1.6%	0.13 [0.05, 0.36]
Zhang 2020	-0.5447	0.1641	10.1%	0.58 [0.42, 0.80]
**Total (95% CI)**			**100.0%**	**0.70 [0.62, 0.80]**

Heterogeneity: Tau² = 0.02; Chi² = 27.12, df = 9 (P = 0.001); I² = 67%
Test for overall effect: Z = 5.13 (P < 0.00001)

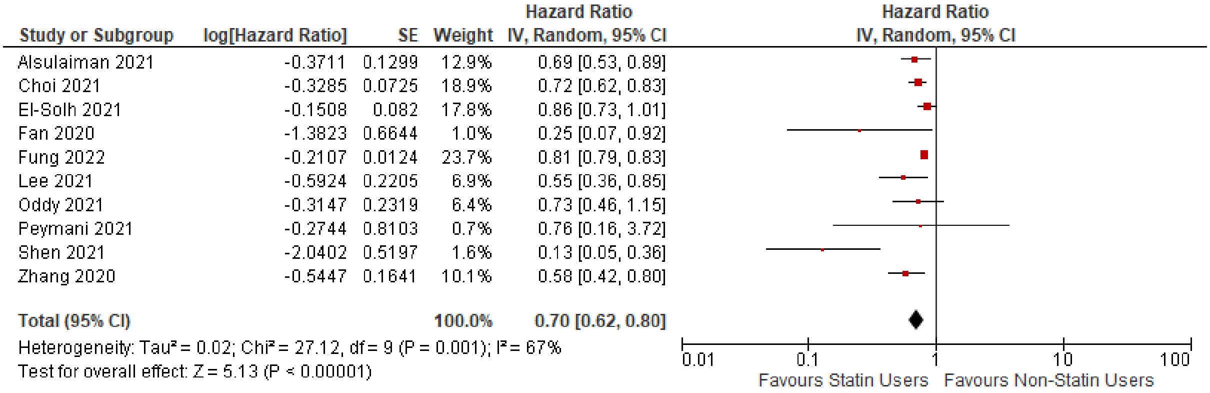

Sensitivity analysis of the mortality odds ratio summary effect by performing separate analysis of studies without (top) or with (bottom) propensity score matching. Our analysis reveal that studies with or without propensity score matching had qualitatively and quantitatively the same effect on the overall estimate. The extent of heterogeneity is improved in the analysis of studies which performed propensity score matching.

Supplementary Figure S6 – Funnel plot and asymmetry analysis for ICU admission odds and hazard ratio

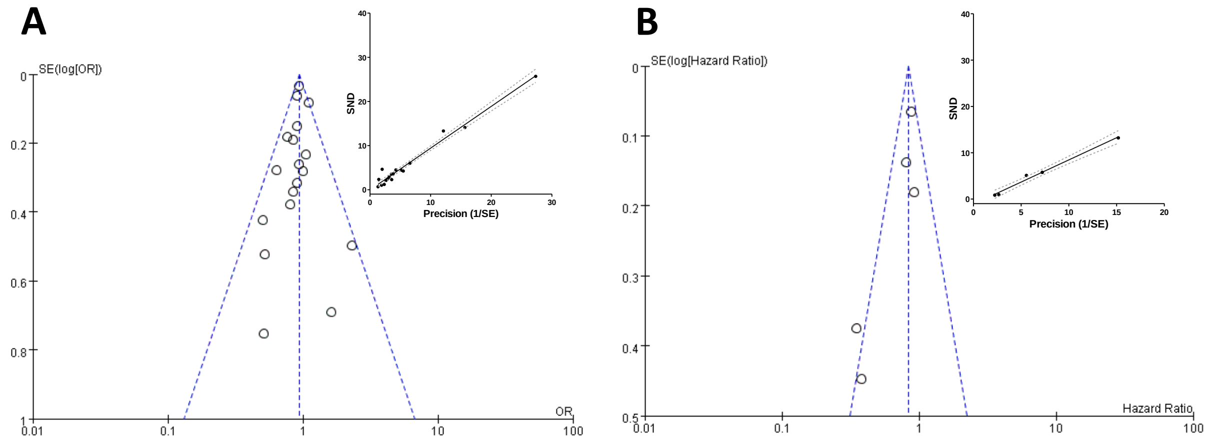

Funnel plots of the standard error of the logarithm of the ICU admission odds (A) or hazard ratio (B). Vertical dotted lines indicate the overall effect and the slanted dotted lines indicate the 95% confidence interval. Inset shows the Egger's regression of the standard normalised difference (SND) against the precision (1/SE). The solid lines are the non-weighted linear regression and the dotted lines indicate the 90% confidence interval of the linear regression. Our analysis reveal no significant asymmetry in the funnel plots (P = 0.87 for OR and P = 0.26 for HR).

Supplementary Figure S7 – Funnel plot and asymmetry analysis for mechanical ventilation odds ratio

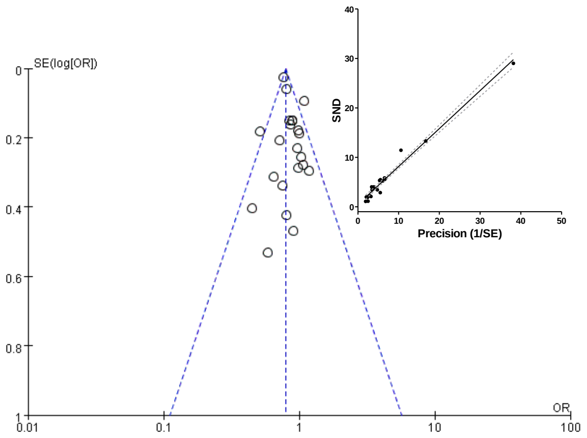

Funnel plots of the standard error of the logarithm of the ICU admission odds ratio. Vertical dotted lines indicate the overall effect and the slanted dotted lines indicate the 95% confidence interval. Inset shows the Egger's regression of the standard normalised difference (SND) against the precision (1/SE). The solid lines are the non-weighted linear regression and the dotted lines indicate the 90% confidence interval of the linear regression. Our analysis reveal no significant asymmetry in the funnel plot for the mechanical ventilation odds ratio (P = 0.29).

Supplementary Figure S8 - Sensitivity analysis of individual study effect on mechanical ventilation hazard ratio

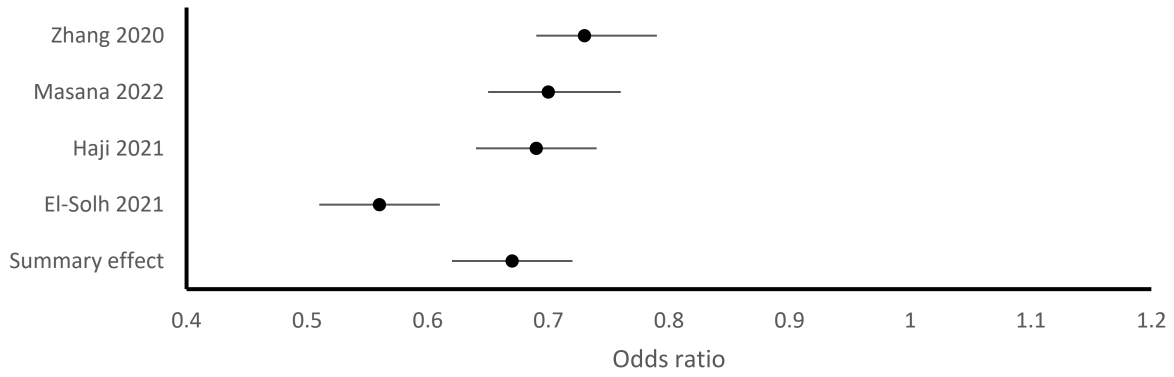

Sensitivity analysis of the data by observing the effect on the overall summary effect (shown at the bottom of the graph) through removing sequential studies. Removal of the El-Solh 2021 study produced a quantitative improvement in the overall estimate of the mechanical ventilation odds ratio.

Supplementary Table S1 – Literature search strategy

**All Fields (PubMed, Web of Science, Scopus) Broad Search (Embase), 2019/12/31 - 2022/04/22**						
	**Search Terms**	**PubMed**	**Embase**	**WOS**	**Scopus**	**Total**
1	statins	7686	27905	4986	20651	61228
2	HMG CoA reductase inhibitors	4148	27148	312	4453	36061
3	atorvastatin or lovastatin or pravastatin or rosuvastatin or fluvastatin	2596	9307	3306	27725	42934
4	statins or HMG CoA reductase inhibitors or atorvastatin or lovastatin or pravastatin or rosuvastatin or fluvastatin	8374	28081	7110	6600	50165
5	COVID 19	252149	257877	255813	449883	1215722
6	coronavirus disease 2019	241717	216515	47423	258583	764238
7	2019 nCoV	159349	62674	2500	76765	301288
8	SARS CoV 2	158885	107865	76872	207743	551365
9	COVID 19 or coronavirus disease 2019 or 2019 nCoV or SARS CoV 2	252149	270412	273450	167883	963894
10	(statins or HMG CoA reductase inhibitors or atorvastatin or lovastatin or pravastatin or rosuvastatin or fluvastatin) AND (COVID 19 or coronavirus disease 2019 or 2019 nCoV or SARS CoV 2)	420	1549	338		
	(statins OR "HMG CoA reductase inhibitors" OR atorvastatin or lovastatin or pravastatin or rosuvastatin or fluvastatin) AND ("covid 19" OR "covid-19" OR "coronavirus disease 2019" OR "2019 ncov" OR "2019-ncov" OR "sars cov 2" OR "sars-cov-2" OR "sars-cov2")				3124	5431

Supplementary Table S2 – Summary of included studies

Study	Type of study	Country	Before /During Hospitalization Statin Use	Type of Statin	Sample Size (Propensity score matched if applicable)		Male (%)	Mean/Median Age (years)*	Diabetes (%)	Outcome
					Statin User	Non-Statin User				
Ageno 2021	Cohort	Italy	Before	N/A	21	91	N/A	N/A	N/A	Mortality
Al-Sulaiman 2021	Cohort	Saudi Arabia	During	Atorvastatin, Rosuvastatin	770 (251)	279 (251)	68.9	61.6 ± 14.8	59.0	Mortality; Mechanical ventilation
An 2020	Cohort	South Korea	Before	N/A	811	7,189	39.9	45.0 ± 19.8	10.0	Mortality
Andrews 2022	Case-control	United States	Before	Atorvastatin	34,474	111,939	51.0	67	40.5	Mortality; ICU admission; Mechanical ventilation
Aparisi 2021	Case-control	Spain	Before	N/A	295	545	50.8	68.2 ± 14.7	19.4	Mortality; ICU admission; Mechanical ventilation
Ayeh 2021	Case-control	United States	Before	Atorvastatin, Pravastatin, Rosuvastatin, Simvastatin	594	3,853	50.0	55.2	18.4	Mortality
Basu 2021	Cohort	United Kingdom	Before	N/A	459	448	54.8	70.9 ± 16.7	45.9	Mortality
Bejan 2021	Cohort	United States	Before	Rosuvastatin, Simvastatin, Atorvastatin	1,333	26,285	39.8	42 ± 20	N/A	Mortality; ICU admission; Mechanical ventilation
Bergqvist 2021	Case-control	Sweden	Before	N/A	169,642	794,234	48.5	64.5	Type 1: 1.15, Type 2: 9.65	Mortality
Bifulco 2021	Cohort	Italy	Before and during	N/A	117	425	63	65.1 ± 13.7	24.0	Mortality
Buenen 2020	Cohort	Netherlands	Before	N/A	209	288	64	72	20.5	Mortality
Bui 2021	Cohort	United States	Before	N/A	8	42	56	70	24	Mortality
Butt 2020	Cohort	Denmark	Before	Atorvastatin, Simvastatin, Rosuvastatin, Pravastatin	843	3,999	47.1	54 (40-72)	8.6	Mortality
Byttebier 2021	Case-control	Belgium	During	N/A	178	571	54.5	69.2	19.6	Mortality
Cabezon 2021	Cohort	Spain	Before	N/A	295	564	50.5	68.1	19.3	Mortality
Cariou 2021	Cohort	France	Before	N/A	1,192	1,257	64.0	70.9 ± 12.5	100	Mechanical ventilation

Castagna 2022	Cohort	United States	Before	N/A	2,718	4,672	51.9	66 (55-77)	55.5	Mortality
Chacko 2021	Case-control	United States	Before	N/A	116	139	51	65.4 ± 15.2	48.2	Mortality; Mechanical ventilation
Cho 2022	Case-control	Korea	Before	Atorvastatin, Rosuvastatin, Others	1,162	6,561	45.3	54	13.0	Mortality
Choi 2021	Cohort	United States	During	Atorvastatin, Rosuvastatin	1,720 (1,445)	3,655 (1,445)	56.6	67.1	High-intensity statin user: 40.0; Low-to-moderate-intensity statin user: 36.8; Non-statin user: 16.1	Mortality; Mechanical ventilation
Chow 2022	Cohort	South Korea	Before	N/A	1,115	3,234	37.0	62.1	18.3	Mortality; ICU admission; Mechanical ventilation
Daniels 2021	Case-control	United States	Before	N/A	763	3,598	55	66 ± 14	40	Mortality; ICU admission; Mechanical ventilation
De Spiegeleer 2021	Cross-section	Belgium	Before	Simvastatin, Atorvastatin, Rosuvastati, Pravastatin, Fluvastatin	185	909	29.1	85.2 ± 8.8	14.4	Mortality
El-Solh 2021	Cohort	United States	Before	Atorvastatin, Rosuvastati, Simvastatin	7,100 (2,692)	7,168 (2,692)	90.7	66 (53-74)	37.6	Mortality; ICU admission; Mechanical ventilation
Espana 2021	Cohort	Spain	Before	N/A	789	2,778	30.3	84.2 ± 10.9	29.7	Mortality
Fan 2020	Case-series	China	Before and during	Atorvastatin, Rosuvastatin, Pravastatin	250 (206)	1,897 (206)	48.5	62	13.6	Mortality; ICU admission; Mechanical ventilation
Fayol 2021	Cohort	France	Before	N/A	58	195	68.8	64.7 ± 16.1	20.2	ICU admission
Fung 2022	Case-control	United States	Before	Atorvastatin, Simvastatin, Lovastatin, Pitavastatin, Pravastatin, Rosuvastatin	187,374	186,925	44.5	N/A	N/A	Mortality
Grasselli 2020	Cohort	Italy	Before	Atorvastatin, Fluvastatin, Lovastatin, Pravastatin, Rosuvastatin, Simvastatin	479	1,236	79.9	63 (56-69)	17.2	Mortality

Greco 2021	Cohort	Italy	Both and during	Atorvastatin, Simvastatin, Pravastatin	51	450	50.7	72 ± 17	13.0	Mortality
Gupta 2021	Case-control	United States	Before	N/A	951 (648)	1,675 (648)	57.0	66	36.9	Mortality; Mechanical ventilation
Haji 2021	Cohort	Iran	During	Atorvastatin	421	570	54.9	61.6 ± 17.0	30.6	Mortality; Mechanical ventilation
Holman 2020	Cohort	United Kingdom	Before	N/A	N/A	N/A	56.0	57.1	100	Mortality
Ikari 2021	Case-control	Japan	Before	N/A	214	479	65	68.3 ± 14.9	38	Mortality; ICU admission; Mechanical ventilation
Kabootari 2022	Cohort	Iran	Before	N/A	111	449	52	61.8 ± 13.4	100	Mortality
Kouhpeikar 2022	Case-control	Iran	During	Atorvastatin	162	421	52.3	61.4 ± 0.9	13.9	Mortality; ICU admission; Mechanical ventilation
Kuno 2022	Case-control	United States	Before and during	Atorvastatin, Fluvastatin, Lovastatin, Pitavastatin, Pravastatin, Rosuvastatin, Simvastatin	2,423 (1,790)	3,672 (1,790)	56.2	65	21.9	Mortality; ICU admission; Mechanical ventilation
Lala 2020	Cohort	United States	Before	N/A	984	1,752	59.6	66.4	26.3	Mortality
Lee 2021	Cohort	Korea	Before	Atorvastatin, Fluvastatin, Lovastatin, Pitavastatin, Pravastatin, Rosuvastatin, Simvastatin	533 (533)	9,915 (1,066)	40.1	44.9 ± 19.8	17.9	Mortality
Li 2022	Case-control	United States	During	Atorvastatin, Fluvastatin, Lovastatin, Pitavastatin, Pravastatin, Rosuvastatin, Simvastatin	3,359 (2,817)	5,538 (2,817)	61.4	63.7	21.1	Mortality; ICU admission; Mechanical ventilation
Lohia 2021 (1)	Cohort	United States	Before	Atorvastatin, Pravastatin, Rosuvastatin, Simvastatin, Lovastatin	454 (233)	560 (233)	52.3	65 (53-73)	42.8	Mortality; ICU admission; Mechanical ventilation
Lohia 2021 (2)	Cohort	United States	Both	Atorvastatin, Pravastatin, Rosuvastatin, Simvastatin	688 (229)	1,156 (229)	53.1	66 (56-73)	44.8	Mortality; ICU admission; Mechanical ventilation
Luo 2020	Cohort	China	During	N/A	55	228	55.1	64	100	Mortality
Ma 2021	Cohort	United Kingdom	Before	N/A	N/A	N/A	46.5	68.6 ± 8.3	13.0	Mortality

Mallow 2020	Cohort	United States	During	N/A	5,313	16,363	52.8	64.9 ± 17.2	42.3	Mortality
Maric 2021	Case-control	United States	Before and during	Atorvastatin, Cerivastatin, Fluvastatin, Lovastatin, Pitavastatin, Pravastatin, Rosuvastatin, Simvastatin	2,297 (2,297)	16,169 (4,594)	47.9	68.4	23.6	Mortality
Masana 2022	Case-control	Spain	Before and during	Atorvastatin, Rosuvastatin	581 (581)	1,576 (581)	57.21	67 (54-78)	23.2	Mortality; ICU admission; Mechanical ventilation
McAlister 2021	Cohort	Canada	Before	Simvastatin, Pravastatin, Atorvastatin, Rosuvastatin	3,978	10,054	44.9	67.7	24.4	Mortality; ICU admission
Mehra 2020	Cohort	United States, Canada, Spain, Italy, Germany, France, United Kingdom, Turkey, China, South Korea, Japan	Before	N/A	860	8,050	60.0	49 ± 16	14.3	Mortality
Meintrup 2021	Cohort	Germany	Before	N/A	147	693	N/A	N/A	N/A	Mortality
Memel 2022	Cohort	United States	Before and during	Atorvastatin, Rosuvastatin, Others	1,285	1,073	57.3	62	34.2	Mortality; ICU admission; Mechanical ventilation
Merzon 2021	Cross-section	Israel	Before	N/A	24	88	55.4	62.9 ± 12.2	42.0	Mortality
Mitacchione 2021	Case-control	Italy	Before	Atorvastatin, Simvastatin, Rosuvastatin, Others	179 (145)	663 (145)	62	64 (61-77)	17	Mortality; ICU admission; Mechanical ventilation
Monserrat Villatoro 2022	Case-control	Spain	Before	Atorvastatin	N/A	N/A	47.9	62.0 (48.0 - 78.0)	17.7	Mortality
Nateghi 2021	Cohort	Iran	Before	Atorvastatin, Rosuvastatin	163	547	57.5	57.0 ± 17.2	30	Mortality; ICU admission
Nicholson 2021	Cohort	United States	Before	N/A	511 (180)	531 (224)	56.8	64 (53-75)	42.5	Mortality; Mechanical ventilation
Oddy 2021	Cohort	United Kingdom	Before	Atorvastatin, Simvastatin	222 (222)	390 (197)	57.8	69.6 ± 17.8	N/A	Mortality; ICU admission
Oh 2021	Cohort	South Korea	Before	N/A	N/A	N/A	N/A	N/A	N/A	Mortality
Pazoki 2021	Cohort	Iran	Before	N/A	107	286	56.2	65.4 ± 11.6	100	Mortality

Peymani 2021	Cohort	Iran	Before and during	Atorvastatin, Rosuvastatin, Simvastatin	75 (75)	75 (75)	58.0	54.7 ± 4.4	21.3	Mortality; Mechanical ventilation
Philipose 2020	Cohort	United Kingdom	Before	Atorvastatin	164	302	59.4	72	Type 1: 1.3; Type 2: 34.1	Mortality
Ramachandran 2020	Cohort	New York	Before	N/A	114	181	54.9	66	44.7	Mortality
Ramos-Rincon 2021	Cohort	Spain	Before	N/A	415	368	52.8	85.8	100	Mortality
Rey 2021	Case-control	Spain	Before	N/A	827	1,364	56.9	68.0 ± 17.8	22.2	Mortality
Rosenthal 2020	Cohort	United States	Before	N/A	11,970	23,332	53.4	63.6 ± 17.7	25.1	Mortality
Saeed 2020	Case-control	United States	During	Atorvastatin, Pravastatin, Rosuvastatin, Simvastatin	1,355	2,897	53	65 ± 16	53	Mortality
Santosa 2022	Cohort	Sweden	Before	N/A	52,128	242,785	48.9	70.9	7.5	Mortality; ICU admission
Shen 2021	Case-control	China	During	N/A	404 (302)	2,729 (302)	50.2	62 ± 19	13.2	Mortality; Mechanical ventilation
Soldevila 2021	Cross-section	Spain	Before	Atorvastatin, Lovastatin, Rosuvastatin, Simvastatin	224	1,082	27.7	86.7	N/A	Mortality
Song 2020	Cohort	United States	Before and during	N/A	123	126	57	62 (51-75)	33.3	Mortality; ICU admission; Mechanical ventilation
Svensson 2021	Case-control	Sweden	Before	N/A	288	798	74.9	62.0 (52.0-70.0)	Type 1: 0.8; Type 2: 25.4	Mechanical ventilation
Terlecki 2021	Cohort	Poland	Before	N/A	269	1,460	51.2	63 (50-75)	25.7	Mortality
Tignanelli 2021	Cohort	United States	Before	N/A	4,286	22,610	56	51.1 (34.6-67.4)	Type 1: 2.84<br>Type 2: 14.5	Mortality
Torres-Pena 2021	Cross-section	Spain	Before and during	N/A	1,130 (934)	1,791 (934)	60.3	72.5	26.3	Mortality; Mechanical ventilation
Umakanthan 2021	Cohort	India	Before	N/A	1,048 (768)	2,204 (768)	55.8	61	47.5	Mortality; Mechanical ventilation
Vahedian-Azimi 2021	Cohort	Iran	Before	Atorvastatin	326	N/A	67.3	54.9 ± 13.8	25.2	Mortality; ICU admission
Vila-Corcoles 2021	Cohort	Spain	Before	N/A	91	445	43.8	65.8 ± 11.3	23.5	Mortality
Volff 2021	Cohort	France	Before	N/A	32	140	73.3	63 (55-71.25)	32	Mortality; Mechanical ventilation

Wander 2021	Cohort	United States	Before	N/A	12,781	23,098	89	60.3 ± 17.0	39	Mortality; ICU admission
Wander 2022	Cohort	United States	Before	Atorvastatin, Fluvastatin, Lovastatin, Pitavastatin, Pravastatin, Rosuvastatin, Simvastatin	69,263	161,891	90	60.9 ± 16.5	36.9	Mortality; ICU admission
Wargny 2021	Cohort	France	Before	N/A	1,282	1,512	63.7	69.7 ± 13.2	100	Mortality
Yetmar 2021	Cohort	United States	Before	Atorvastatin, Rosuvastatin, Fluvastatin, Lovastatin, Pitavastatin, Pravastatin, Simvastatin	500	795	55.4	60	30.1	Mortality
Zhang 2020	Case-control	China	During	Atorvastatin, Rosuvastatin, Simvastatin, Pravastatin, Fluvastatin, Pitavastatin	1,219 (861)	12,762 (3,444)	48.9	61.5	16.3	Mortality; ICU admission; Mechanical ventilation
Zhong 2020	Cohort	China	Before	N/A	11	115	44.4	66	32.5	Mortality

Supplementary Table S3 – Newcastle-Ottawa Scale assessment of study quality

Study	Selection of Cohorts	Comparability of Cohorts	Outcome	Total Score	Quality
Ageno 2021	★★★		★★★	6	Good
Alsulaiman 2021	★★★	★	★★★	7	Good
An 2020	★★★★		★★	6	Good
Andrews 2022	★★★★	★	★★★	8	Good
Aparisi 2021	★★★★	★	★★★	8	Good
Ayeh 2021	★★★★		★★★	7	Good
Basu 2021	★★★★		★★★	7	Good
Bejan 2021	★★★★		★★★	7	Good
Bergqvist 2021	★★★★	★	★★★	8	Good
Bifulco 2021	★★★★	★	★★★	8	Good
Buenen 2020	★★★★		★★★	7	Good
Bui 2021	★★★		★★★	6	Good
Butt 2020	★★★★		★★★	7	Good
Byttebier 2021	★★★★	★★	★★★	9	Good
Cabezon 2021	★★★★		★★★	7	Good
Cariou 2021	★★★	★★	★★★	8	Good
Castagna 2022	★★★★		★★★	7	Good
Chacko 2021	★★★★	★	★★★	8	Good
Cho 2022	★★★★		★★★	7	Good
Choi 2021	★★★★	★	★★★	8	Good
Chow 2022	★★★★	★	★★★	8	Good
Daniels 2021	★★★★	★	★★★	8	Good
De Spiegeleer 2021	★★★	★	★★★	7	Good
El-Solh 2021	★★★★	★	★★★	8	Good

Espana 2021	★★★★		★★★	7	Good
Fan 2020	★★★★	★★	★★★	9	Good
Fayol 2021	★★★★		★★★	7	Good
Fung 2022	★★★★		★★★	7	Good
Grasselli 2020	★★★		★★★	6	Good
Greco 2021	★★★★	★	★★★	8	Good
Gupta 2021	★★★★	★★	★★★	9	Good
Haarhaus 2021	★★★		★★★	6	Good
Haji 2021	★★★★	★★	★★★	9	Good
Holman 2020	★★★	★	★★★	7	Good
Ikari 2021	★★★	★★	★★★	8	Good
Kabootari 2022	★★★		★★★	6	Good
Kouhpeikar 2022	★★★★	★	★★★	8	Good
Kuno 2022	★★★★	★★	★★★	9	Good
Lala 2020	★★★★		★★★	8	Good
Lee 2021	★★★★	★★	★★★	9	Good
Li 2022	★★★★	★★	★★★	9	Good
Lohia 2021	★★★★	★★	★★★	9	Good
Lohia 2021 (2)	★★★★	★★	★★★	9	Good
Luo 2020	★★★		★★★	6	Good
Ma 2021	★★★		★★★	6	Good
Mallow 2020	★★★★		★★	6	Good
Maric 2021	★★★★	★	★★★	8	Good
Masana 2022	★★★★	★	★★★	8	Good
McAlister 2021	★★★★	★	★★★	8	Good
Mehra 2020	★★★★		★★★	7	Good

Meintrup 2021	★★★★		★★★	7	Good
Memel 2022	★★★★		★★★	7	Good
Merzon 2021	★★★★		★★★	7	Good
Mitacchione 2021	★★★★	★★	★★★	9	Good
Monserrat Villatoro 2022	★★★★		★★★	7	Good
Nateghi 2021	★★★★	★	★★★	8	Good
Nicholson 2021	★★★★		★★★	7	Good
Oddy 2021	★★★★		★★★	7	Good
Oh 2021	★★★★	★★	★★	8	Good
Pazoki 2021	★★★		★★★	6	Good
Peymani 2021	★★★★	★	★★★	8	Good
Philipose 2020	★★★★		★★★	7	Good
Ramachandran 2020	★★★★		★★★	7	Good
Ramos-Rincon 2021	★★★		★★★	6	Good
Rey 2021	★★★★	★	★★★	8	Good
Rosenthal 2020	★★★★		★★★	7	Good
Saeed 2020	★★★★	★	★★★	8	Good
Santosa 2022	★★★★	★	★★★	8	Good
Shen 2021	★★★★	★★	★★★	9	Good
Soldevila 2021	★★★		★★★	6	Good
Song 2020	★★★★	★★	★★	8	Good
Svensson 2021	★★★		★★★	6	Good
Terlecki 2021	★★★★		★★★	7	Good
Tignanelli 2021	★★★★		★★	6	Good
Torres-Pena 2021	★★★★	★★	★★★	9	Good
Umakanthan 2021	★★★★	★★	★★★	9	Good

Vahedian-Azimi 2021	★★★★		★★	6	Good
Vila-Corcoles 2021	★★★		★★★	6	Good
Volff 2021	★★★		★★★	6	Good
Wander 2021	★★★★		★★	6	Good
Wander 2022	★★★★	★	★★★	8	Good
Wargny 2021	★★★		★★★	6	Good
Yetmar 2021	★★★★	★	★★★	8	Good
Zhang 2020	★★★★	★	★★★	8	Good
Zhong 2020	★★★		★★★	6	Good
